# Rates of Never Receiving Cervical Cancer Screening Among US Women, 2005-2023

**DOI:** 10.1001/jamanetworkopen.2026.31550

**Published:** 2026-08-27

**Authors:** Kalyani Sonawane, Haluk Damgacioglu, Trisha L. Amboree, Marvella E. Ford, Ketki N. Borse, Brian Orr, Gweneth Lazenby, Ana Patrica Ortiz, Ashish A. Deshmukh

**Affiliations:** 1 Department of Public Health Sciences, College of Medicine, Medical University of South Carolina, Charleston; 2 MUSC Hollings Cancer Center, Charleston, South Carolina; 3 Department of Obstetrics and Gynecology, College of Medicine, Medical University of South Carolina, Charleston; 4 Cancer Control and Population Sciences Program, University of Puerto Rico Comprehensive Cancer Center, San Juan, Puerto Rico

## Abstract

This cross-sectional study examines trends in the prevalence of never having been screened for cervical cancer from 2005 to 2023 and assesses trends in cervical cancer incidence by age and stage at diagnosis to determine whether incidence patterns correspond with trends in nonscreening.

## Introduction

Despite being preventable, cervical cancer continues to cause avoidable morbidity and mortality when women are not reached by screening programs.^1^ Identifying women who have never been screened for cervical cancer is critical because this group captures the population entirely missed by preventive care, revealing the deepest shortcomings in reach. This is especially important for the newly eligible group aged 21 to 29 years, for whom early engagement with screening sets the trajectory for lifelong screening behavior, whereas identifying never-screened women aged 30 to 65 years reveals missed prevention opportunities during the years of highest cervical cancer risk.^2^ We examined trends in the prevalence of never having been screened for cervical cancer from 2005 to 2023 and characterized sociodemographic disparities in nonscreening. We additionally assessed trends in cervical cancer incidence by age and stage at diagnosis to determine whether incidence patterns correspond with trends in nonscreening.

## Methods

The Medical University of South Carolina institutional review board deemed this cross-sectional study exempt from review and waived the requirements for informed consent because publicly available data were used. This study followed the STROBE reporting guidelines.

Data from the National Health Interview Survey (NHIS) cycles 2005, 2008, 2010, 2013, 2015, 2018, 2019, 2021, and 2023, which included a cervical cancer screening questionnaire, were analyzed.^3^ The proportions of women who reported never having undergone cervical cancer screening at the time of each survey year were estimated for groups aged 21 to 29 years and 30 to 65 years; denominators were corrected for hysterectomy. Human papillomavirus (HPV) vaccination status (available in NHIS cycles 2008, 2010, 2015, 2018, and 2019) was also ascertained for women aged 21 to 29 years.

Using US Cancer Statistics data,^4^ we examined cervical cancer incidence by age (30-64 and ≥65 years) and SEER summary stage (localized, regional, or distant) during 2001 to 2023. The older age groups allow sufficient time for cancers to manifest among women who were unscreened earlier in life. Age-standardized cervical cancer incidence rates and corresponding SEs were calculated using the hysterectomy-corrected population at risk. A detailed description of data sources and study criteria is available in the eAppendix in Supplement 1.

Joinpoint regression examined temporal trends and provided estimates of annual percentage change (APC) and average APC (AAPC). SEER*Stat software version 8.4.5 (National Cancer Institute) was used to examine time trends.^4^ Disparities in never-screening were estimated using the most recent survey year (2023) to fit logistic regression models for calculating prevalence ratios. Statistical significance was tested at 2-sided *P* < .05. Analyses were performed from January to April 2026 using SAS version 9.4 (SAS Institute), and adjusted for the complex survey design.

## Results

The final analytical sample included cervical cancer screening-eligible women from 9 NHIS cycles aged 21 to 29 years (23 502 women [22%]) and 30 to 65 years (82 760 women [78%]) at the time of the survey. The HPV vaccination rate among the group aged 21 to 29 years ranged from 5.7% (142 women) in 2008 to 54.0% (941 women) in 2019. From 2005 to 2023, the proportion of women never screened for cervical cancer between ages 21 and 29 years increased from 12.5% (3.5 million) to 31.9% (6.2 million) (AAPC, 5.2%; 95% CI, 4.3%-7.5%) (Figure, panel A), with a pronounced increase (APC, 7.9%; 95% CI, 6.0%-14.4%) from 2010 to 2023. Among women aged 30 to 65 years, the proportion increased from 5.3% (4.3 million) to 14.8% (9.2 million) (AAPC, 6.8%; 95% CI, 3.1%-12.0%) (Figure, panel B).

**Figure.**
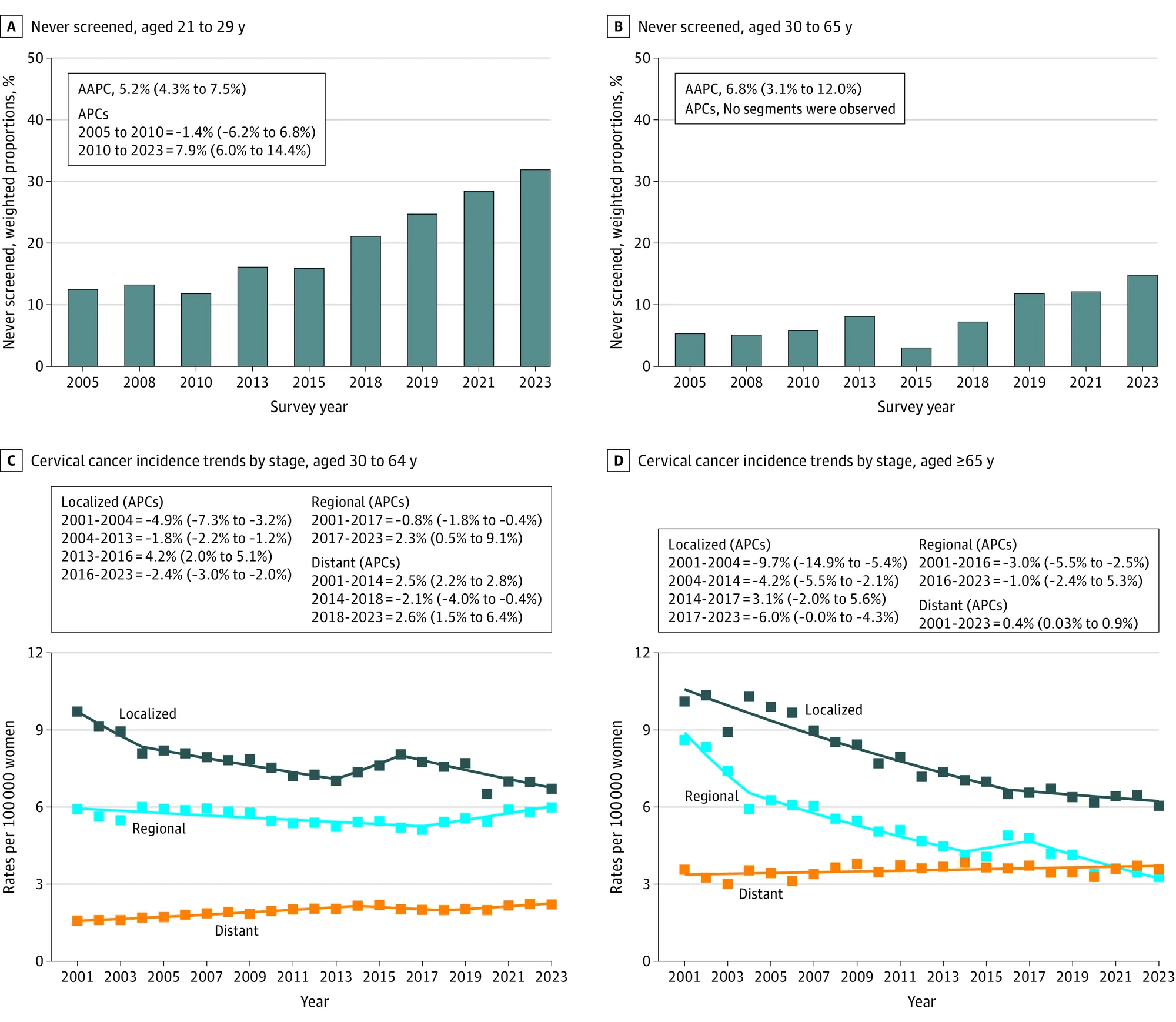
Bar Graphs and Line Graphs of Trends in Proportion of US Women Who Never Underwent Cervical Cancer Screening and Cervical Cancer Incidence by Stage at Diagnosis by Age Group A and B, Bar graphs show the proportion of US women who reported never being screened for cervical cancer at the time of the survey. Weighted proportions of women never screened for cervical cancer and human papillomavirus (HPV) vaccination rates were estimated using National Health Interview Survey survey weights. The proportion of women aged 21 to 29 years who reported that they were HPV vaccinated (≥1 dose) was 5.7% in 2008, 12.6% in 2010, 26.1% in 2013, 42.3% in 2018, and 54.0% in 2019. C and D, Line graphs show age-standardized cervical cancer incidence by stage at diagnosis US Cancer Statistics data, corrected for hysterectomy. Trends were examined using Joinpoint regression to estimate annual percentage change (APC) and average annual percentage change (AAPC). Numbers in parentheses are 95% CIs.

Trends in cervical cancer incidence (APC) are shown in panels C (ages 30-64 years) and D (ages ≥65 years) of the Figure. Among women aged 30 to 64 years, regional-stage and distant-stage incidence each increased significantly by more than 2% per year after 2017. Among women aged 65 years and older, distant-stage incidence has increased since 2001.

The 2023 NHIS included data for 8901 women aged 21 to 65 years (mean [SE] age, 40.5 [0.16] years) who were predominantly White individuals (5187 women [55.8%]) with some college education (2418 women [29.4%]), private insurance (5635 women [62.5%]), and urban status (7777 women [88.3%]) living in the South (3227 women [36.9%]). Among women aged 21 to 29 years, never-screening was highest among Asian women (44.3% [95% CI, 32.07%-56.58%]) compared with 28.8% (95% CI, 25.07%-32.57%) among non-Hispanic White women, and among women with a high school degree (39.0%; 95% CI, 33.00%-45.07%) or a college or associate’s degree (35.0%; 95% CI, 29.45%-40.51%) vs those with a master’s or doctoral degree (17.1%; 95% CI, 10.59%-23.69%) (Table). Findings were consistent in the 30- to 65-year-old age group, with additional disparities noted by race and ethnicity, education, insurance, and region.

**Table.** Women Who Never Underwent Cervical Cancer Screening by Sociodemographic Characteristics

Characteristic	Women aged 21-29 y		Women aged 30-65 y	
	Weighted % (95% CI)	PR (95% CI)^a^	Weighted % (95% CI)	PR (95% CI)^a^
Race and ethnicity^b^				
American Indian or Alaskan Native	38.3 (16.69-59.87)	1.33 (0.59-1.48)	15.5 (7.33-23.60)	1.32 (0.70-1.99)
Asian	44.3 (32.07-56.58)	1.48 (1.23-1.49)	26.1 (21.36-30.76)	2.27 (2.13-2.30)
Hispanic	37.5 (32.14-42.86)	1.20 (0.98-1.37)	25.6 (22.77-28.50)	1.79 (1.59-1.94)
Non-Hispanic Black	33.9 (25.69-42.01)	1.13 (0.83-1.37)	18.6 (15.21-22.88)	1.56 (1.29-1.81)
Non-Hispanic White	28.8 (25.07-32.57)	1 [Reference]	8.8 (7.72-10.00)	1 [Reference]
Other	16.5 (6.66-26.17)	0.55 (0.25-1.06)	9.4 (4.55-14.24)	1.02 (0.58-1.64)
Education				
Grades 1-11	32.1 (17.46-46.68)	1.50 (0.80-1.74)	28.1 (22.56-33.31)	1.57 (1.23-1.83)
General Educational Development or equivalent	26.5 (12.13-40.93)	1.50 (0.70-1.90)	24.8 (18.24-31.38)	1.82 (1.44-2.04)
High school graduate	39.0 (33.00-45.07)	1.76 (1.44-1.86)	20.5 (17.93-23.03)	1.71 (1.41-1.99)
College or associate’s degree	35.0 (29.45-40.51)	2.14 (1.47-2.58)	13.3 (11.44-15.14)	1.42 (1.12-1.76)
Bachelor’s degree	26.4 (21.73-31.10)	1.44 (0.98-1.85)	10.6 (8.91-12.22)	1.19 (0.93-1.50)
Master’s or doctoral degree	17.1 (10.59-23.69)	1 [Reference]	8.1 (6.42-9.90)	1 [Reference]
Insurance				
Private	30.3 (6.74-33.86)	1.13 (0.72-1.50)	11.1 (9.80-12.42)	0.82 (0.60-1.09)
Public	31.6 (25.60-37.58)	1.06 (0.69-1.41)	18.4 (15.62-21.13)	0.98 (0.72-1.29)
Other	29.9 (0.77-39.11)	1 [Reference]	16.7 (12.93-20.39)	1 [Reference]
Uninsured	41.8 (32.77-50.91)	1.27 (0.89-1.48)	31.8 (27.32-36.27)	1.40 (1.12-1.61)
Income-to-poverty ratio				
Below poverty level	35.0 (26.73-43.34)	0.95 (0.62-1.27)	23.5 (19.98-27.08)	0.78 (0.56-1.05)
<2	30.9 (25.20(36.73)	0.86 (0.57-1.18)	20.3 (17.67-23.02)	0.92 (0.72-0.21)
<3	35.3(28.91-41.69)	1.05 (0.74-1.32)	18.5 (15.63-21.37)	1.03 (0.79-1.31)
<5	31.3 (25.72-36.78)	0.90 (0.63-1.18)	12.6 (10.67-14.55)	1.89 (0.65-1.19)
≥5	28.2 (22.00-34.47)	1 [Reference]	9.0 (7.55-10.54)	1 [Reference]
Area of residence				
Urban	31.9 (28.93-34.97)	0.95 (0.69-1.23)	15.0 (13.77-16.28)	1.10 (0.84-1.39)
Rural	31.6 (23.99-39.11)	1 [Reference]	12.9 (10.00-15.82)	1 [Reference]
Region				
Midwest	28.1 (21.89-34.29)	0.80 (0.55-1.11)	11.5 (9.40-13.52)	1.14 (0.88-1.44)
Northeast	35.9 (28.92-43.05)	1.08 (0.76-1.44)	14.8 (11.70-17.82)	1.30 (1.01-1.62)
South	31.0 (26.65-35.39)	0.92 (0.72-1.12)	17.1 (14.99-19.13)	1.27 (1.04-1.51)
West	33.8 (28.03-39.63)	1 [Reference]	14.1 (11.91-16.25)	1 [Reference]

Abbreviation: PR, prevalence ratio.

^a^ Logistic regression models simultaneously adjusting for sociodemographic characteristics were used to estimate PRs of never having been screened for cervical cancer.

^b^ Race and ethnicity was self-reported by survey participants. Other includes Mexican American, other Hispanic, non-Hispanic American Indian or Alaskan Native, or other races, including multiracial.

## Discussion

This cross-sectional study found that in the US, the proportion of women never screened for cervical cancer increased by more than 5% per year during 2005 to 2023. By 2023, nearly 1 in 3 women aged 21 to 29 years and 1 in 7 aged 30 to 65 years had never been screened. Some gap is expected among newly age-eligible women, but the near-doubling in the proportion of unscreened younger women is concerning. Findings from the current study, together with a 2019 NHIS analysis, indicate that among women aged 21 to 29 years, nearly 8 million (45.5%) remain unvaccinated, and almost one-third of these women have also never been screened, highlighting substantial missed opportunities for both primary and secondary prevention.^5^ Meanwhile, regional-stage and distant-stage cervical cancer incidence each increased by more than 2% per year in recent years (after 2017) among women aged 30 to 65 years. These increases are troubling, particularly when viewed in the context of the proportion of women who have never been screened for cervical cancer over the last 2 decades.

The overall increase in never receiving cervical cancer screening is further compounded by inequities across demographic and geographic subgroups. Structurally vulnerable subgroups, including minoritized racial groups, uninsured women, and those residing in the Southern states, have a greater likelihood of never having cervical cancer screening. These disparities are especially concerning given that late-stage diagnoses and mortality are higher among minoritized racial groups, uninsured women, and women living in the South.^6,7,8^ A limitation of this study is the self-reported nature of the screening data, which is subject to recall bias, and the lack of availability of HPV status in the NHIS and cancer registries. Although we present incidence data alongside screening prevalence, our objective is not to establish causal associations but to generate hypotheses for future causal work.

This study reports a troubling increase in never-screened women and overlap between populations at greatest risk and those least likely to receive preventive screening. The findings underscore a growing gap in cervical cancer prevention among young women and during midlife and highlight the need for targeted strategies to address structural barriers and improve screening uptake.

## Notes

Supplement 1. eAppendix. Data Sources and Sample Identification

Supplement 2. Data Sharing Statement
