# Supplementary material for: Rates of Never Receiving Cervical Cancer Screening Among US Women, 2005-2023
Source: JAMA Netw Open. 2026 Aug 27;9(8):e2631550. doi: 10.1001/jamanetworkopen.2026.31550 (PMC13522957; doi:10.1001/jamanetworkopen.2026.31550)
Supplement: Supplementary file 1 [file jamanetwopen-e2631550-s001.pdf]

Supplemental Online Content

Sonawane K, Damgacioglu H, Amboree TL, et al. Rates of never receiving cervical cancer screening among US women, 2005-2023. *JAMA Netw Open*. 2026;9(8):e2631550. doi:10.1001/jamanetworkopen.2026.31550

eAppendix. Data Sources and Sample Identification

This supplemental material has been provided by the authors to give readers additional information about their work.

© 2026 Sonawane K et al. *JAMA Network Open*.

eAppendix. Data Sources and Sample Identification

Never screened for cervical cancer

Data from the National Health Interview Survey (NHIS) cycles 2005, 2008, 2010, 2013, 2015, 2018, 2019, 2021, and 2023, which included a cervical cancer screening questionnaire, were analyzed. To examine time trends in never receiving cervical cancer screening, age-eligible women between 21 and 65 years at the time the NHIS was conducted were identified. Women who had reportedly undergone a hysterectomy and had missing information on cervical cancer screening were excluded. Two screening-eligible age groups (21-29 and 30-65 years old) were identified. The outcomes of interest were never-initiated screening. Never screened was defined using the question "*Have you EVER HAD a Pap smear or Pap test?"* during 2005-2015 and "*Have you EVER HAD a test to check for cervical cancer*?" in 2018, 2019, 2021, and 2023. Human papillomavirus (HPV) vaccination status (available in NHIS cycles 2008, 2010, 2015, 2018, and 2019) was also ascertained for women aged 21-29 years. Vaccination status: "Have you *EVER* received an HPV shot or vaccine?" Because women aged 30-65 years were largely age ineligible to receive HPV vaccination at the time of the survey, analysis of HPV vaccination status was restricted to the 21–29-year-old age group.

Cervical cancer incidence and incidence-based mortality

We analyzed the US Cancer Statistics dataset from the Centers for Disease Control and Prevention's (CDC) National Program of Cancer Registries and the National Cancer Institute's (NCI) Surveillance, Epidemiology, and End Results (SEER) Program on cases of cervical cancer diagnosed from 2001 to 2019 covering 97% of the US population for all 50 states and the District of Columbia (cases diagnosed in Mississippi and Indiana were not included as they did not meet USCS reporting standards during the entire study period).[5] Data from 2020 were not included for the incidence analysis due to significant disruptions to healthcare delivery during the COVID-19 pandemic and subsequent changes in registry operation or changes in data collection, which affected cancer screening, diagnosis, and treatment and may have compromised the completeness of cancer incidence reporting delays.[6] We identified malignant and microscopically confirmed cases of cervical cancer using International Classification of Diseases Oncology-3 (ICD-O-3) codes C53.0-C53.9 and histology codes 8010–8671 and 8940–8941. We excluded cases diagnosed by autopsy or death certificate only. To classify the stage at diagnosis, we used a merged summary stage variable that utilizes SEER Summary Stage 2000, Derived SEER Summary Stage 2000, and Summary Stage 2018, depending on the year of diagnosis, to categorize cancer cases based on the extent of disease spread. The stage at diagnosis was categorized as: localized (confined to the cervix), regional (spread outside the cervix area to nearby structures or lymph nodes), distant (spread to distant parts of the body), and unknown stage. Cervical cancer incidence rates were corrected using population-level hysterectomy prevalence rates. Hysterectomy prevalence was estimated using data from the Behavioral Risk Factor Surveillance System (BRFSS).[7] Using logistic regression, we calculated survey-weighted prevalence estimates based on age groups, years, and their interaction, using BRFSS data. Adjusted prevalence rates were derived for each age and year group. To estimate the population at risk, women with a history of hysterectomy were excluded from the

© 2026 Sonawane K et al. *JAMA Network Open*.

denominator. To accurately reflect cases where a hysterectomy was performed for cervical cancer treatment, those individuals were added back into the adjusted denominator.

Statistical Analysis

Descriptive statistics (weighted population estimates and proportions) were used to examine the proportion of women never screened in the two age groups. Descriptive statistics (proportions) were used to examine the proportion of women aged 21-29 years who were HPV vaccinated. We quantified calendar trends in the proportion never screened and calculated the Annual Percentage Change (APC) and Average Annual Percentage Change (AAPC) using the NCI's Joinpoint Regression Program. Statistical significance was tested at 2-sided $P<.05$. All NHIS analyses were performed using SAS®, Cary, NC, and were adjusted for strata, clusters, and weights to account for the complex survey design.

© 2026 Sonawane K et al. *JAMA Network Open*.
