# Supplementary material for: Rates of Never Receiving Cervical Cancer Screening Among US Women, 2005-2023
Source: JAMA Netw Open. 2026 Aug 27;9(8):e2631550. doi: 10.1001/jamanetworkopen.2026.31550 (PMC13522957; doi:10.1001/jamanetworkopen.2026.31550)
Supplement: Supplementary file 2 [file jamanetwopen-e2631550-s002.pdf]

Data Sharing Statement

Sonawane. Rates of Never Receiving Cervical Cancer Screening Among US Women, 2005-2023. *JAMA Netw Open*. Published August 28, 2026.
doi:10.1001/jamanetworkopen.2026.31550

Data
Data available: Yes
Data types: Deidentified participant data and cancer registry data
How to access data: Data can be accessed at https://www.cdc.gov/nchs/nhis/index.html and https://seer.cancer.gov/data/
When available: With publication

Supporting Documents
Document types: None

Additional Information
Who can access the data: Anyone
Types of analyses: For research purposes.
Mechanisms of data availability: Upon NHIS and NPCR approval
